# Lung contusion after extracorporeal shock wave lithotripsy for pancreatic stones: A case report

**DOI:** 10.1097/MD.0000000000030063

**Published:** 2022-08-12

**Authors:** Jin-Hui Yi, Dan Wang, Hui Chen, Zhao-Shen Li, Liang-Hao Hu

**Affiliations:** Department of Gastroenterology, Changhai Hospital, Naval Medical University, Shanghai, China.

**Keywords:** case report, complications, lung contusion, pancreatic extracorporeal shock wave

## Abstract

**Background:**

Pancreatic extracorporeal shock wave lithotripsy (P-ESWL) is recommended as the first-line treatment for large pancreatic stones. While complications such as post-P-ESWL pancreatitis, bleeding, infection, steinstrasse, and perforation have been reported in the past 30 years, lung contusion has never been reported. The present case demonstrates lung contusion as a complication after P-ESWL.

**Methods:**

A 48-year-old man was admitted to our department due to painful chronic pancreatitis with pancreatic duct stones. Computed tomography revealed normal lungs. P-ESWL was performed. The shock wave head contacted with right upper quadrant and the path of shock wave was at a 45° angle to the ventral midline. After P-ESWL, multiple patchy high-density shadows in the lower lobe of right lung were found, which was normal before P-ESWL. The patient had no symptoms of lung injury.

**Results and Conclusion:**

Laboratory studies revealed elevated D-dimer from 0.33 to 0.74 ug/mL, which was consistent with abnormal clotting of lung contusion. Chest computed tomography showed slight pleural effusion. Considering the interval between 2 X-rays was only 3 hours, we inferred that lung contusion was related to P-ESWL. The patient displayed stable vital signs, therefore, no specific interventions were conducted. Three days after P-ESWL, endoscopic retrograde cholangiopancreatography was performed and the lung shadows were partially absorbed. Considering the location of shock wave head, it was possible to cause lung contusion in lower lobe of right lung. More than 10,000 P-ESWL therapeutic sessions had been performed in our center since 2010, and it is the first case about lung contusion as a complication. It is also the first report to describe lung contusion after P-ESWL. Although the patient was asymptomatic, it should raise awareness of clinicians.

## 1. Introduction

Pancreatic stone is typical change of chronic pancreatitis with an incidence of more than 90%.^[1]^ Stones obstructing the pancreatic duct may lead to hypertension of pancreatic duct and ischemia of pancreatic parenchymal, which would induce abdominal pain and accelerate the deterioration of pancreatic function. Therefore, symptomatic pancreatic stone should be treated. Pancreatic extracorporeal shock wave lithotripsy (P-ESWL) was recommended by guideline as the first-line treatment for large pancreatic stones.^[2]^ Complications of P-ESWL such as pancreatitis, bleeding, and steinstrasse have been reported in the past 30 years. Here, we present a patient developed lung contusion after P-ESWL.

## 2. Case presentation

**Figure 1.**
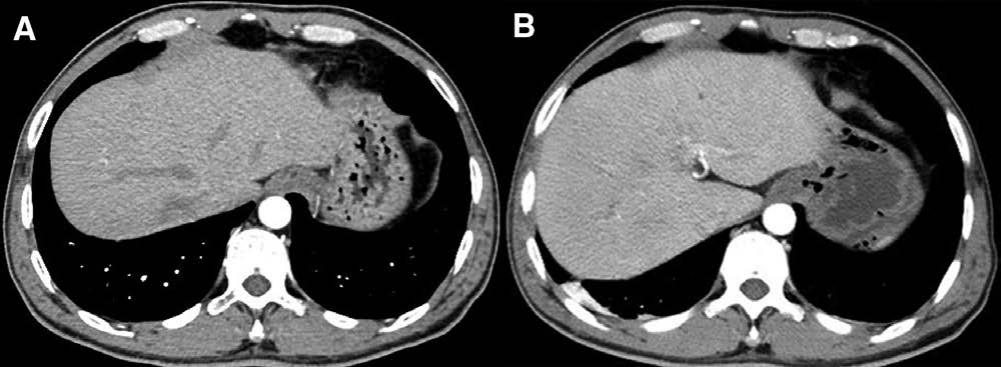
CT scan. (A) CT revealed normal lower lobe of the both lungs before the first P-ESWL. (B) Chest CT showed slight pleural effusion after the second P-ESWL. CT = computed tomography, P-ESWL = pancreatic extracorporeal shock wave lithotripsy.

A 48-year-old man was admitted to our department due to intermittent upper abdominal pain with radiating pain in lower back. Computed tomography revealed upstream dilation of pancreatic duct with radiopaque stones in the pancreatic body, and no abnormality was observed in the lower lobe of both lungs (Fig. 1A). The result confirmed the diagnosis of chronic pancreatitis. Upon admission, complete blood count, basic metabolic panel, blood clotting tests, and chest X-ray were performed and all the results were within normal range. There was no contradiction of P-ESWL. With the informed consent of the patient, P-ESWL treatment was performed on the patient using the third-generation lithotripter (Delta Compact II, Dornier MedTech, Wessling, Germany) with the patient in a 30°-right supine position. The shock wave head contacted with right upper quadrant and the path of shock wave was at a 45° angle to the ventral midline.^[3]^ Up to 5000 shock waves were delivered per therapeutic session at an intensity of 6 (16,000 kV) on a scale of 1 to 6 with a frequency of 120 shocks/min. During the procedure, flurbiprofen and remifentanil were combined for analgesia by intravenous infusion. Two sessions of P-ESWL were performed, and digital radiographic X-ray system (MD-8000A, Canon, Tokyo, Japan) was used to assess the size of stone before and after each session of P-ESWL.

**Figure 2.**
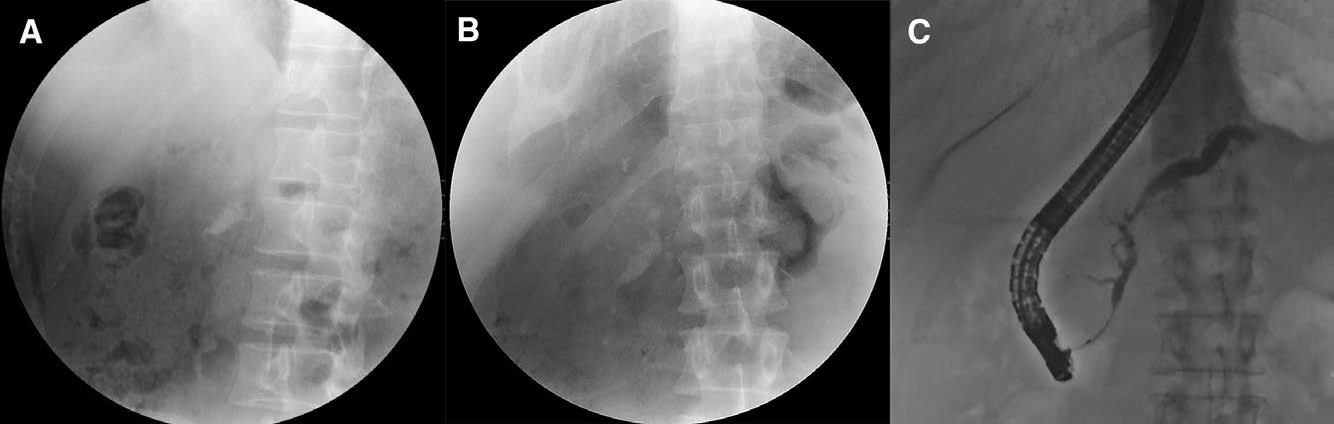
X-ray scan. (A) The X-ray image before second P-ESWL showed normal lower lobe of right lung. (B) The X-ray image after second P-ESWL showed patchy high-density shadows in the lower lobe of right lung (arrow). (C) The X-ray image before endoscopic retrograde cholangiopancreatography showed lung shadows were partially absorbed (arrow). P-ESWL = pancreatic extracorporeal shock wave lithotripsy.

However, after the second P-ESWL, patchy high-density shadows in the lower lobe of right lung were found, which did not exist before the second P-ESWL (Fig. 2A and B). The patient had no symptoms such as abdominal pain, emesis, diarrhea, cough, hemoptysis, or hypoxemia. Laboratory studies revealed a hemoglobin drop from 136 to 119 g/L, elevated serum amylase of 174 U/L, elevated D-dimer from 0.33 to 0.74 ug/mL, normal carbon dioxide combining power, and serum procalcitonin. Chest computed tomography showed slight pleural effusion (Fig. 1B) caused by lung contusion. Considering the interval between 2 X-rays was only 3 hours, we thought lung contusion was related to P-ESWL. As the patient had stable vital signs and no complaints of discomfort, no specific interventions were conducted. Three days later, endoscopic retrograde cholangiopancreatography was performed to clear pancreatic duct and the lung shadows were observed to be partially absorbed (Fig. 2C). The patient was discharged after 2 days with no discomfort. During follow-up, no symptoms such as fever, cough, or hemoptysis occurred till the lung shadows were completely absorbed 5 months later.

## 3. Discussion and Conclusion

Although many studies have proved the safety and efficiency of P-ESWL,^[4,5]^ it is still important to pay attention to postoperative complications. The mechanisms of complications may be as follows: firstly, energy of the shock wave is partially released before reaching the target stone, which will damage tissues on the pathway of shock wave; secondly, slight movement of pancreas caused by respiration motion results the unstable location of the target. Due to the individual variation in the anatomy location of pancreas, complications of P-ESWL are varied and difficult to predict. The overall complication rate was 6.7%, including pancreatitis, bleeding, infection, steinstrasse, and perforation.^[6]^ Rare complications have also been reported, such as hepatic subcapsular hematoma,^[3]^ splenic hematoma,^[7]^ colonic hematoma,^[8]^ large mesenteric hematoma,^[9]^ and pancreaticobiliary fistula.^[10]^ Lung contusion has been reported after urinary ESWL,^[11]^ but to our knowledge, lung contusion has never been reported after P-ESWL. Considering the location of shock wave head, it was possible to cause lung contusion in lower lobe of right lung.

Pulmonary hemorrhage has been reported in animal models evaluating urinary ESWL-related lung contusion. In mouse models, both pneumocytes and endothelial cells were damaged by shock waves, resulting a direct communication between vessel lumina and alveolar spaces, leading to hemoptysis.^[12]^ In rabbit models, Senyucel et al^[13]^ found that shock waves caused epithelial desquamation and peribronchial congestion. Our patient was asymptomatic except for elevated D-dimer, which was consistent with abnormal clotting of lung contusion. Lung contusion may be ignored without related symptoms and the incidence of this complication may be underestimated. Prompt diagnosis of lung contusion before any severe clinical manifestations occurred is of significant importance as life threatening hypoxemia following urinary ESWL has been reported.^[14]^

No specific change needs to be made during the P-ESWL since lung contusion caused by shock wave is moderate and rarely happened. Treatment experience is lacking. If patient condition is stable, infection prevention and vital signs monitoring wound be the first choice. When symptoms are severe, positive end-expiratory pressure ventilation and fluid resuscitation might be needed.

The damage to the lung is generally mild as the energy released by scattered waves is limited. Therefore, diagnosis of lung contusion for patients with no specific symptoms is likely to be missed. More than 10,000 P-ESWL therapeutic sessions have been conducted since 2010 in our center, and it is the first case about lung contusion. It is also the first case report to describe lung contusion after P-ESWL. Although the patient was asymptomatic, it should raise awareness of clinicians.

## Author contributions

Jin-Hui Yi, Dan Wang, and Hui Chen participated in the acquisition, analysis, and interpretation of data, as well as in the manuscript drafting. Zhao-Shen Li and Liang-Hao Hu contributed to the conception, design, and data interpretation, as well as revised the manuscript for important intellectual content. All authors have read and approved the manuscript, and ensure that this is the case.
